# Evaluation of an HPV vaccine toolkit to improve OB/GYN discussion of HPV vaccination

**DOI:** 10.1016/j.puhip.2024.100473

**Published:** 2024-02-05

**Authors:** Sarah Simko, Teodocia Maria Hayes-Bautista, Erica Givens

**Affiliations:** a University of California at Riverside, Riverside, CA, USA; b Adventist Health White Memorial Medical Center, Los Angeles, CA, USA

**Keywords:** HPV vaccination, Provider discussion, Catch-up Immunization, Multi-component, Cancer Prevention, Behavior change

## Abstract

**Background:**

HPV (Human Papillomavirus) vaccination is a safe, effective method to prevent HPV-associated disease. Racial-ethnic disparities in HPV vaccination exist, which could lead to widening gaps in cervical cancer mortality. Provider discussion of HPV vaccination has been shown to be a primary factor for increasing vaccination rates. The objective of this study is to assess provider discussion of HPV vaccination pre and post implementation of an intervention, named the HPV Vaccine Toolkit, in an Obstetrics and Gynecology (OB/GYN) clinic in Boyle Heights, Los Angeles.

**Study design and methods:**

This quality improvement study occurred over four cycles of development. Its design was guided by the Theory of Planned Behavior. The toolkit components included dot phrases (pre-written phrases to speed documentation), educational posters, electronic health record prompts, HPV vaccine referral guides, and educational sessions. Chart audits and pre- and post-providers surveys were performed between 2019 and 2021 to assess for an increase in provider discussion of the HPV vaccine, as well as to evaluate the various components of the toolkit.

**Results:**

Provider discussion increased over the four cycles of this intervention, with HPV vaccination discussion documented in 15 % of patients in 2019, 19 % of patients in 2020 and 47 % of patients in 2021. Gaps identified included limited discussion of vaccination at postpartum visits. Provider uncertainty of where to refer patients for the HPV vaccine decreased following the intervention.

**Conclusion:**

Discussion of HPV vaccination is an important preventative strategy that can be overlooked in OB/GYN clinics. Implementation of multicomponent strategies can increase provider discussion of HPV vaccination status, although barriers to discussion remain. Improved counseling on HPV vaccination could have significant impacts on reducing HPV-related disease.

## 1 What this study adds

• HPV vaccination can be overlooked in OB/GYN clinic, especially at postpartum visits.
• Multi-component interventions can be effective at increasing provider discussion of HPV vaccination.
• Barriers to provider discussion of HPV vaccination include lack of time, forgetting, and competing priorities.

## 2 Implications for Policy and Practice

• Practices should address low vaccination rates through multiples strategies with interval evaluation of vaccine uptake.
• Strategies should aim at increasing ease of behavioral change for providers by attempting to address specific clinical barriers.

## 3 Introduction

Human papillomavirus (HPV) is a sexually transmitted infection that affects up to 80 % of women during their lifetime [1]. HPV is responsible for approximately 91 % of cases of cervical cancer and is also associated with vulvar, vaginal, oropharyngeal, and anal cancer [2]. Compared with Caucasian women, Hispanic women experience 70 % higher cervical cancer prevalence and 52 % higher mortality rate due to cervical cancer [3].

HPV vaccination is an important preventative strategy. The HPV vaccine prevents HPV types that cause up to 90 % of cervical cancers [4]. The vaccine is recommended by the CDC for boys and girls at age 11–12, with catch up vaccination through age 26. Patient-provider discussion is recommended for patients aged 27–45 [5].

Despite proven safety and efficacy of the HPV vaccine, vaccination rates remain well below the Healthy People 2030 goal of 80 %. In 2021, the Center for Disease Control and Prevention (CDC) reported 63.8 % of girls in the United States between 13 and 17 had completed the HPV vaccine series. While rates of HPV vaccination are increasing among adolescents, the rate lags behind coverage rates for other routine vaccinations [6]. Additionally rates of vaccination among young adults are much lower, with only 51.5 % of women between the age 19–26 having received at least one dose of the vaccine in 2017 [7]. This is a key age group for vaccination because among the 14 million new HPV infections that occur each year, about 50 % occur between the ages of 15–24 [8].

Racial ethnic disparities in HPV vaccination exist, with lower rates of HPV vaccination initiation and completion in Hispanic women [9,10]. These gaps in HPV vaccination could lead to a widening of the existing disparities in cervical cancer mortality in the United States unless actions are taken now to increase vaccination rates.

Physician recommendation has been found to be one of the most important factors for increasing HPV vaccine uptake [11]. Studies on interventions to improve provider recommendation of the HPV vaccine have included the use of clinical decision prompts in outpatient clinics [12]. Additionally, interventions aimed at improving provider communication about the HPV vaccine through training and coaching have shown some success [13,14]. Fewer of these interventions have been performed in Obstetrics and Gynecology (OB/GYN) clinic settings.

Given that physician behavior change can be difficult due to structural barriers that arise in the clinic, we looked to theoretical frameworks to guide our intervention. Various theoretical frameworks and models such as the Social Cognitive Theory, the Health Belief Model, and the Theory of Reasoned Action have helped us understand what influences the behavior of patients' in regards to HPV vaccine uptake [15,16]. These models can also help predict components that can impact provider behavior change; for example, a study by Millstein showed the utility of the Theory of Planned Behavior in predicting provider behavior [17]. Icek Ajzen's Theory of Planned Behavior assumes that people's intentions are influenced by their attitudes, social norms, and perceived behavior control, or ease of behavioral change, and that people's intentions lead to behavior changes [18]. This theoretical framework was chosen to guide the development of this intervention.

### 3.1 Objectives

The primary objective of our study was to increase the frequency of provider discussion and recommendation of the HPV vaccine through a multi-component intervention.

The secondary objective of our study was to evaluate an HPV Vaccine Toolkit developed to facilitate provider-patient discussion of HPV vaccination, as well as provider recommendation and documentation.

### 3.2 Local Problem

An audit at an OB-GYN clinic in 2019 of 87 electronic medical records of patients aged 9–26 revealed that only a small percentage 15 % (13/87) of providers had discussed the HPV vaccine.

### 3.3 Rationale

This intervention was guided by the theory that ease of behavioral modifications, through structural changes and shifting attitudes on vaccination, could drive a change in physician behavior. By addressing voiced barriers raised by providers and emphasizing the importance of vaccination in an at-risk patient population, we hoped to drive behavioral change by encouraging provider intention to vaccinate in the clinic to reduce disparities in cervical cancer.

## 4 Methods

### 4.1 Context

Our improvement intervention study was conducted at an outpatient OB/GYN clinic. The clinic is located in the greater East Los Angeles area of California. It provides comprehensive OB/GYN care to about 2000 patients a month. The patient demographic served is predominantly Hispanic, with some patients preferring the Spanish language to communicate. Many are Medicaid-eligible. The clinic collaborates with an OB/GYN residency program and hosts both a resident clinic and an attending clinic. It is accredited by the Accreditation Council for Graduate Medical Education (ACGME).

### 4.2 The study population

The study population consisted of OB/GYN residents and attendings.

### 4.3 Intervention

Creating awareness and motivating adoption of preventative measures like the HPV vaccine can be complex. This study focused on physician behavior. The goal of the intervention was to create interest and perceived behavioral control through: 1) environmental cues and educational tools (posters in the waiting room and exam rooms) [Appendix A]; 2) reminders to discuss the HPV vaccine (electronic health record (EHR) note template reminders); 3) facilitators for physician documentation of HPV vaccine discussion (dot phrases); and 4) resources to help physicians inform patients on where to get vaccinated (HPV vaccine referral guide) [Appendix A]. The elements of our intervention were named the “HPV Vaccine Toolkit.”

The roadmap to creating the HPV Vaccine Toolkit consisted of multiple steps. Informal meetings with clinic office managers and medical assistants were held to gather information about the flow of clinic. Physician provider surveys were performed to assess baseline knowledge of the HPV vaccine and current practices and barriers. Provider interactive educational sessions were held to educate providers on the HPV vaccine and impress the importance of vaccination [19]. EHR audits were performed to assess the rates of provider discussion of the vaccine in the clinic. Given that the clinic did not offer the HPV vaccine, community sources for the HPV vaccine were identified to create the HPV vaccine referral guide. Finally, the components of the HPV Vaccine Toolkit were evaluated to assess for utility.

### 4.4 Study Design

The impact of our intervention was evaluated using a quasi–experimental design. There was no randomization of providers, no control group, and no effort to control for possible confounding variables. A pre/post survey of physicians [Appendix B] and an audit of patients' electronic records were performed to observe if there was an increase in physician discussion and recommendation of the HPV vaccine. Specific questions were included to determine the percentage of visits the providers' recalled using components of the toolkit in the post-survey. This approach, we believed, could help us establish if the observed outcome was due to the effects of the HPV Vaccine Toolkit. Our study was deemed to be IRB exempt by the Loma Linda Institutional Review Board.

### 4.5 Measures

Electronic records of patients aged 9–26 seen at the clinic for either a well-woman annual visit (WWE), new gynecology visit, postpartum visit (PP), or colposcopy appointment were audited for evidence of provider-patient discussion of the HPV vaccine, the recommendation to get the HPV vaccine, or history that the patient had received an HPV vaccine. These were the visit types that were selected as most beneficial and conducive to including a discussion of HPV vaccination.

### 4.6 Iterative development

**Fig. 1:**
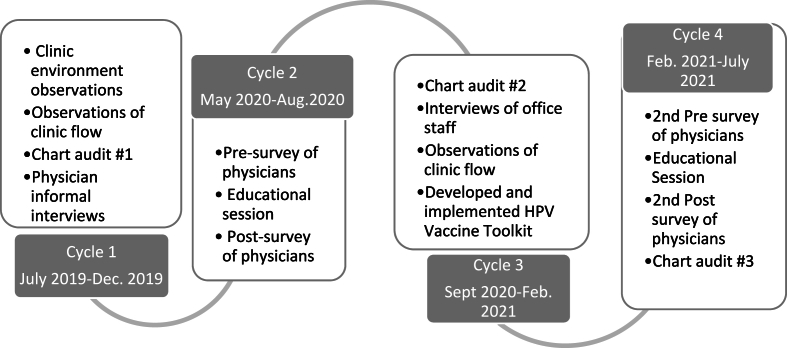
Cycles of development.

This intervention was developed over three years through multiple cycles of observations, provider surveys, and electronic medical record audits (Fig. 1).

#### 4.6.1 Cycle 1 (July 2019–December 2019)

##### 4.6.1.1 Quantitative

Electronic medical records were audited from July 2019 to August 2019. Preliminary results showed low rates of provider discussion and recommendation of HPV vaccination.

##### 4.6.1.2 Qualitative

Informal physician interviews and focused discussions on HPV vaccination noted the HPV vaccine is often something that gets forgotten.

“I just forget, too many competing demands.”

“There are just too many things to talk about.”

##### 4.6.1.3 Observations

1. Residents are provided a checklist reminder to discuss preventative measures during well woman exams, including the HPV vaccine. Discussion of the HPV vaccine rarely gets marked as discussed.
2. Patient information handouts on the HPV vaccine are not available in the clinic.
3. The HPV vaccine is not available in the clinic for administration.

#### 4.6.2 Cycle 2 (may 2020–August 2020)

##### 4.6.2.1 Educational sessions

An interactive lecture was held by one of the authors on the current CDC recommendations for HPV vaccination, known disparities in HPV vaccination rates, and guidelines for best practices on how to discuss HPV vaccination with patients.

##### 4.6.2.2 Quantitative

Pre- and post-surveys were administered to assess baseline HPV vaccine knowledge and facilitators and barriers to providers’ discussion and recommendation of the HPV vaccine. Seven providers responded to the pre-survey; five responded to the post survey.

Generally, physicians believed a provider's recommendation influences patient's decision to get vaccinated. However, the survey showed that baseline knowledge of cervical cancer disparities was limited - none selected Hispanic patients as having the highest incidence of cervical cancer.

Barriers identified by physicians were: lack of time, patient's low health literacy, confusion with recommendations, and uncertainty of where to refer patients for the HPV vaccination.

Recommendations from physicians included a need to know more about insurance coverage for the HPV vaccine and request to make the HPV vaccine accessible on site.

“Get the vaccine in the clinic.”

“A lot of patients don’t have Gardasil covered by their insurance and the cost is a huge barrier to them receiving the vaccine. If we could carry some of the discounted/free vaccines, I think we could vaccinate more people.”

#### 4.6.3 Cycle 3 (September 2020–February 2021)

##### 4.6.3.1 Quantitative

A second chart review was performed from July 2020 to August 2020 to assess for increase in vaccine discussion after the didactic intervention. Review of the frequency of provider discussion in 2019 compared to 2020 showed minimal increase.

##### 4.6.3.2 Contextual factors

Informal discussions were held with clinic manager to explore the possibility of getting access to the HPV vaccine on-site; however, the cost of the HPV vaccine and the difficulty of storing the vaccine in the clinic were prohibitive.

##### 4.6.3.3 The patient clinic flow

The patient clinic flow was studied and optimal placement of educational posters were identified as the intake window in the waiting room and in patient exam rooms.

Additionally, because medical assistants' communication with patients and providers is a critical component of the clinic flow, and medical assistants were already in the practice of handing the patients’ intake form to providers with other forms, this was identified as an appropriate opportunity to introduce the HPV vaccine referral guides. Medical assistants were asked to include the HPV vaccine referral guides with the patient intake forms for patients 9–26 with the goal of reminding providers to discuss the HPV vaccine.

##### 4.6.3.4 Toolkit development

The elements included in the HPV Vaccine Toolkit were created as a response to physicians’ voiced needs and concerns.

HPV vaccine posters were created using information on vaccine purpose, safety, and efficacy, obtained through the CDC, to provide a clear message to patients that the HPV vaccine is a safe, effective method that can prevent cancer. These were placed in patient exam rooms and in the waiting room.

The HPV vaccine referral form was created to educate both providers and patients on where the HPV vaccine could be accessed. QR codes were imbedded to links where patients could sign up to get vaccinated through local pharmacies, Planned Parenthood or local health departments. This referral infographic form included the HPV dosing schedule and possible insurance coverage of cost.

Dot phrases were created to streamline provider documentation. These were created by researching current standards for HPV vaccination by the CDC on age of vaccination, dosing, and special considerations, including immunocompromised state, pregnancy, breastfeeding. Dot phrases on the safety of the HPV vaccine and side effects of the vaccine were also created. These phrases were accessible to providers through shared dot phrases in the clinic EHR.

EHR reminders were placed in the New Gynecology, Well Woman Exam, Postpartum, and Colposcopy note templates. This was done through discussion with the clinic manager.

#### 4.6.4 Cycle 4 (February 2021–July 2021)

##### 4.6.4.1 2nd educational session

In February 2021, a second interactive lecture was held. Seventeen physicians (5 attendings and 12 residents) attended. Emphasis was placed on HPV vaccination rate disparities in Hispanic patient populations and the influence of a physicians’ recommendation on patient vaccine uptake.

##### 4.6.4.2 Quantitative

A pre-survey [Appendix B] was administered to the providers at the time of this second educational session to assess for utility of the various components, feedback and barriers to discussion of the HPV vaccine.

##### 4.6.4.3 Informal discussions

Informal discussion with clinic staff was held to seek their feedback.

“I like the QR codes, not every patient likes paper.”

“I like the info-graphs”

##### 4.6.4.4 Implementation of the HPV vaccine toolkit

One month before the HPV Vaccine Toolkit was implemented, an email communication was sent out to all outpatient clinic physicians recommending and detailing the various components of the HPV Vaccine Toolkit.

The clinic manager and medical assistants were also informed of the toolkit and encouraged to hand the HPV vaccine referral guide to providers with the patient intake form for patients aged 9–26.

The HPV Vaccine Toolkit was implemented in March 2021.

##### 4.6.4.5 Quantitative

A third electronic medical record audit was performed from July to August 2021.

##### 4.6.4.6 Qualitative

A postsurvey [Appendix B] was administered in June 2021 to assess how useful the various components were in practice for providers, how often they used each component, and to again seek feedback and assess remaining barriers to discussion.

In the post-survey, physicians were asked about their discussion of the HPV vaccine in the postpartum period.

“I am typically focused on other issues at postpartum visits.”

“Not something I think about discussing at a pp visit.”

“[I] Forget, too many other things to discuss.”

Findings from the chart review and provider surveys were reviewed.

## 5 RESULTS

### 5.1 Chart review

There were 87 patients identified in the first audit from July to August 2019, 85 in the second chart audit from July to August 2020, and 53 in the third chart audit from July to August 2021. Physician discussion and recommendation of HPV vaccine was 15 %, 19 %, and 47 % from 2019 to 2021, respectively (Table 1).

**Table 1:** Provider discussion and recommendation by year

Percentage	2019 (n=87)		2020 (n=85)		2021 (n=53)	
Response	Number	Percentage	Number	Percentage	Number	Percentage
**Not discussed**	74	85	69	81	28	53
**Discussed**	13	15	16	19	25	47

**Fig. 2:**
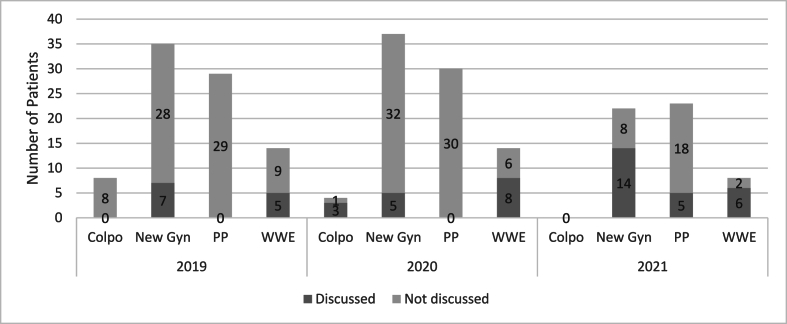
Number of Patients who Experienced Provider Discussion of HPV Vaccination by Visit Type Abbreviations: Colpo: Colposcopy, New Gyn: New Gynecology, PP: Postpartum, WWE: Well Woman Exam.

When assessing provider discussion by visit type, HPV vaccine discussion was noted in gynecologic visits more frequently that in postpartum visits (Fig. 2). In new gynecology and well woman visits, discussion rates increased from 20 % to 36 % in 2019 to 64 % and 75 % in 2023, respectively. Postpartum visits increased more modestly from 0 % in 2019 to 22 % in 2021. Discussion at colposcopy visits could not be assessed due to low numbers.

### 5.2 Provider survey

The most dramatic change reported in the provider survey was a significant drop in feeling uncertain of where to refer patients for an HPV vaccine, i.e., from 71 % reporting uncertainty in the pre-survey to 0 % reporting uncertainty in the post-survey. Provider's sense of the priority of educating patients on HPV vaccination in the clinic increased from 71 % in the pre-group to 100 % in the post-group.

Intended behavior statements in the pre-survey were compared to post-survey statements of actual behavior, which showed less than expected use of info-graphs, referral guide, and dot phrases (Table 2). The majority of providers (70 %) stated the poster was useful in counseling patients, and 70 % stated that receiving the HPV vaccine referral guide with their patient intake form reminded them to discuss the vaccine. Only 20 % of providers stated that they utilized the dot phrases for more than half of their patient visits.

**Table 2:** Provider Survey results

Item	Pre (n=17) 17 providers, including 5 attendings and 12 residents	Post (n=10) 10 providers, including 4 attendings and 6 residents
Laminated Infographic/Poster	The majority of providers (94 %) stated that they planned to use the laminated infographic.	50 % of providers stated they used the laminated infographic at greater than half of their patient encounters for patients aged 26 years or younger. 70 % of providers stated that the poster was helpful in counseling patients.
HPV Vaccine Referral Guide	100 % of providers stated receiving the HPV vaccine referral form with the patient's intake form would help them remember to discuss the HPV vaccine.	50 % of providers stated they received the referral form with the patients' intake form at more than half of the patient visits. 70 % of providers stated that receiving the form helped remind them to discuss the HPV vaccine.
Dot Phrases	The majority of providers (88 %) stated that they planned to use the dot phrases.	20 % of providers stated they used the dot phrases for more than half of their patient visits 40 % of providers stated that the dot phrases were useful.
Confidence	Most providers stated they felt confident in their ability to counsel patients on the HPV vaccine (94 %) and felt up to date on the current recommendations (88 %).	The majority of providers stated they felt confident in counseling patients on the HPV vaccine (90 %) and felt up to date on current recommendations (100 %).
Priority	71 % of providers stated that educating women about the HPV vaccination was a priority in the clinic.	All providers stated that educating women about HPV vaccination was a priority in the clinic.
Barriers	The top concerns expressed by providers in the pre-survey were uncertainty of where to send patients for the vaccine (71 %), lack of time (47 %), and competing priorities (47 %).	The top concerns expressed included forgetting to mention (90 %), competing priorities (70 %), and lack of time (70 %). New barriers that arose included uncertainty that the patient would follow up to receive the vaccine (20 %).
Recommendation	Recommendations by providers included obtaining the vaccine in the clinic and providing information about vaccine cost and insurance coverage for patients.	Feedback included obtaining the vaccine in the clinic, periodic reinforcement and reminders in the clinic, and obtaining access to the California Immunization Registry for the clinic.

## 6 DISCUSSION

From 2019 to 2021, an increase was seen in the percent of OB/GYN visits that included a physician HPV vaccination recommendation. Additionally, provider uncertainty with where to refer patients for the HPV vaccine drastically decreased after the intervention and provider attitudes towards the importance of HPV vaccination increased. Despite these findings, barriers in the clinic persist, including lack of time and competing priorities, particularly at postpartum visits.

Prior studies have assessed interventions targeting providers to increase HPV-vaccination rates with mixed findings. A systemic review by Mavundza, et al. demonstrated increased initiation of the HPV vaccine with provider prompts, provider training, funding**,** and multicomponent interventions; however, only interventions including provider prompts, funding and multicomponent interventions were found to increase completion of the vaccine series [20]. Although our study assessed provider discussion rather than vaccine uptake, we similarly saw minimal change in discussion of the vaccine following provider education alone between 2019 and 2020. Multicomponent techniques can be more successful in increasing vaccination rates for the HPV vaccine, as well as for other vaccines [21,22].

Our intervention strove to incorporate multi-component changes into an OB-GYN resident clinic. This study is unique because it developed through stages with specific attention to the reported barriers described by providers and clinic staff. The Theory of Planned Behavior informed the development of our intervention as we desired to 1) change the norm in the clinic by creating a culture of vaccination through repeated discussion and visual reminders, 2) supply the provider with tools that would make discussion and recommendation of the HPV vaccine easier, 3) change provider attitudes to prioritize HPV vaccination through provider education. Our findings support the importance of iterative development in quality improvement endeavors and providing providers with tools to support behavior changes in the clinic over provider education alone.

While improvements in provider discussion and attitude towards HPV vaccination were noted, differences were found between intended physician behavior with their actual experience during our evaluation of the components of the toolkit. Our interest in the comparison of intended provider behavior and actual behavior was again guided by the Theory of Planned Behavior, as studies have shown that provider intention can lead to behavior change [23]. Physicians’ intention to use specific tools was less than their actual experience using these tools. Lack of time and competing priorities were identified as barriers that may have contributed to this difference.

Additionally, our chart audit showed that postpartum women did not receive any recommendation for the HPV vaccine during the first two years of the intervention. Lake et al., 2022 observed that physicians were more likely to recommend vaccination to young, unmarried women compared to older married women [24]. In our third year, postpartum women did begin to receive the recommendation, indicating some change in behavior. In the provider survey, providers reported that they often forget to discuss the HPV vaccine at postpartum visits or prioritize other topics. These sentiments are reflected in the literature [25]. Increasing discussion of vaccination at postpartum visits is an important area to focus future efforts.

Evaluating the results of this intervention, there is room for further improvement. Areas of focus include working with other care providers, including medical assistants to promote discussion of the HPV vaccine, working to ensure the HPV vaccine referral guides are available with the intake form, and improving provider training in the use of dot phrases. Larger scale structural changes include obtaining access to the HPV vaccine and to the California Immunization Registry (CAIR), so that HPV vaccine uptake can be directly assessed. Recent efforts to partner with the hospital pharmacy have led to the stocking the HPV vaccine for our patients to access. While cost and limited resources for vaccine storage can be a limiting factor to obtaining the HPV vaccine in a low-resource clinic setting, other routes to access can include partnering with local pharmacies or nearby clinics.

While this quality improvement study has multiple strengths, limitations of this study include lack of randomization or control group; therefore, interpretation of our findings is limited by the potential for confounding factors. Vaccine uptake was also unable to be evaluated, as the clinic did not have access to CAIR, and patient reported vaccine uptake was limited by provider documentation.

Strengths of this quality improvement study include multiple cycles of both quantitative and qualitative evaluation and iterative development of the intervention with targeted design based on analysis of clinic flow. Additionally, this was a low-cost intervention that can be replicated in other low-resource academic OB/GYN clinic.

The findings of this study have implications for public health measures that can be implemented in other practice settings. When attempting to address low HPV vaccination rates, clinical practices should utilize multi-component strategies aimed at increasing ease of behavioral change for providers by attempting to address specific clinical barriers. Interval evaluation of vaccine uptake to assess for intervention efficacy should be performed with modifications as necessary.

## 7 CONCLUSION

This study assessed the use of a multi-component toolkit that was developed through multiple cycles of evaluation to increase provider discussion and recommendation of the HPV vaccine in an OB/GYN resident clinic. This intervention aimed to target various components that are thought to be important in affecting provider intention, with the ultimate goal of affecting behavior change. These findings are significant in that they may signal to other OB/GYNs the importance of discussing the HPV vaccine with patients in this catch-up period and provide low-cost strategies that can be utilized and re-evaluated in other settings.

While HPV vaccine efficacy is highest in adolescents, vaccine efficacy has been shown to be very effective in young adults and is recommended by the CDC through the age of 26, with targeted recommendations up to age 45. Given women in this age group are often most likely to see an OB/GYN, it is essential that we prioritize protocols in OB/GYN clinics to prevent unvaccinated women from falling through the cracks, especially for patients at increased risk of developing HPV-associated cancers.

## Declaration of competing interest

The authors declare that they have no known competing financial interests or personal relationships that could have appeared to influence the work reported in this paper.
