# Supplementary material for: Evaluation of an HPV vaccine toolkit to improve OB/GYN discussion of HPV vaccination
Source: Public Health Pract (Oxf). 2024 Feb 5;7:100473. doi: 10.1016/j.puhip.2024.100473 (PMC10867569; doi:10.1016/j.puhip.2024.100473)
Supplement: Supplementary file 1 [file mmc1.docx]

**Appendices:**

**Appendix A:**

**HPV Vaccine Educational Posters (in English and Spanish):**


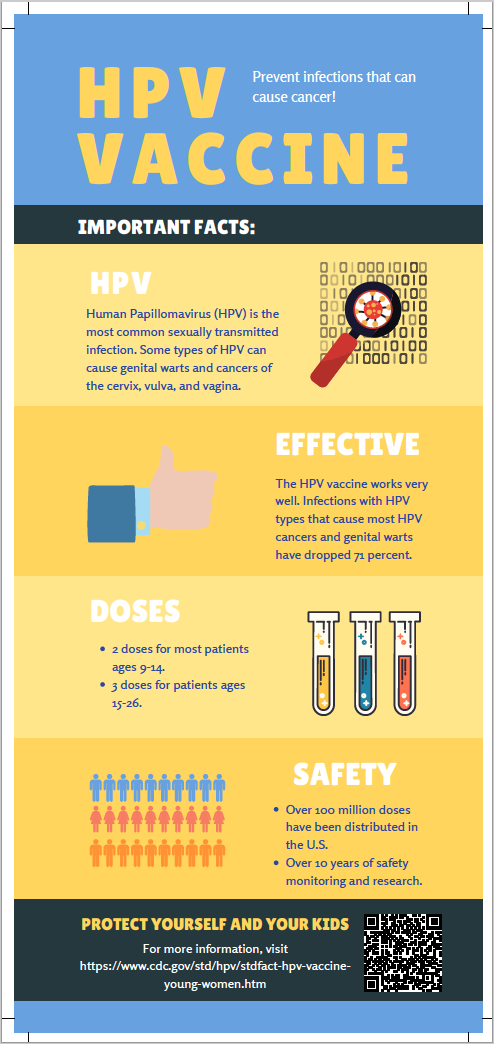

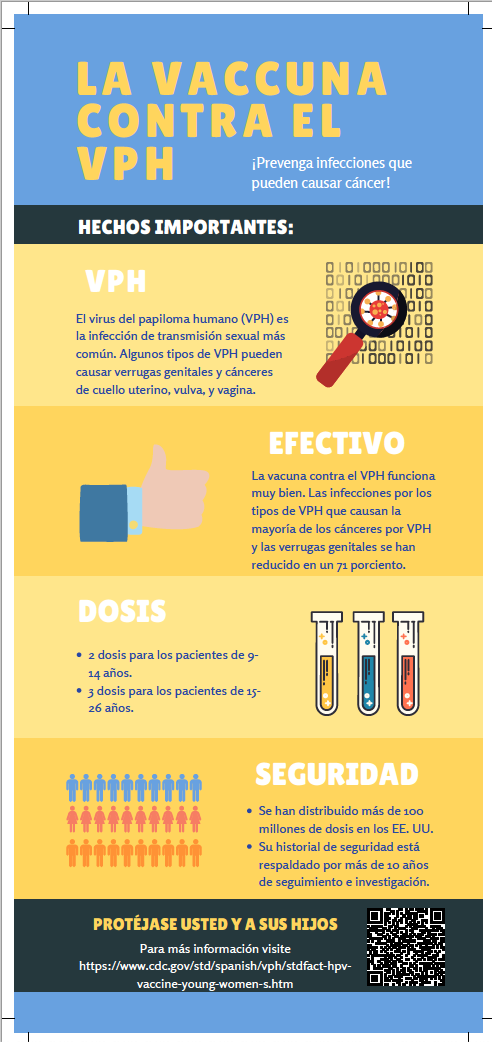


**HPV Vaccine Referral Guide (in English and Spanish):**


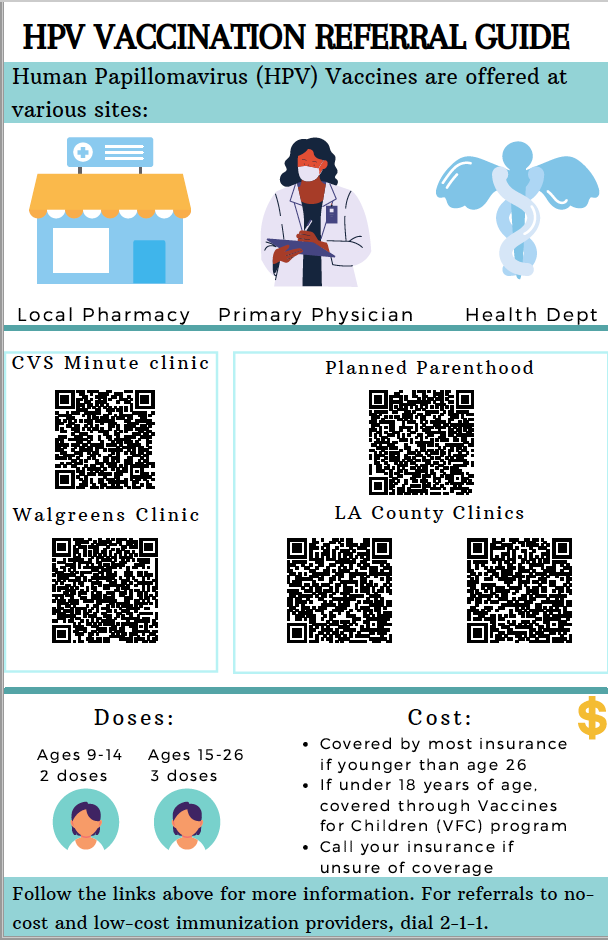

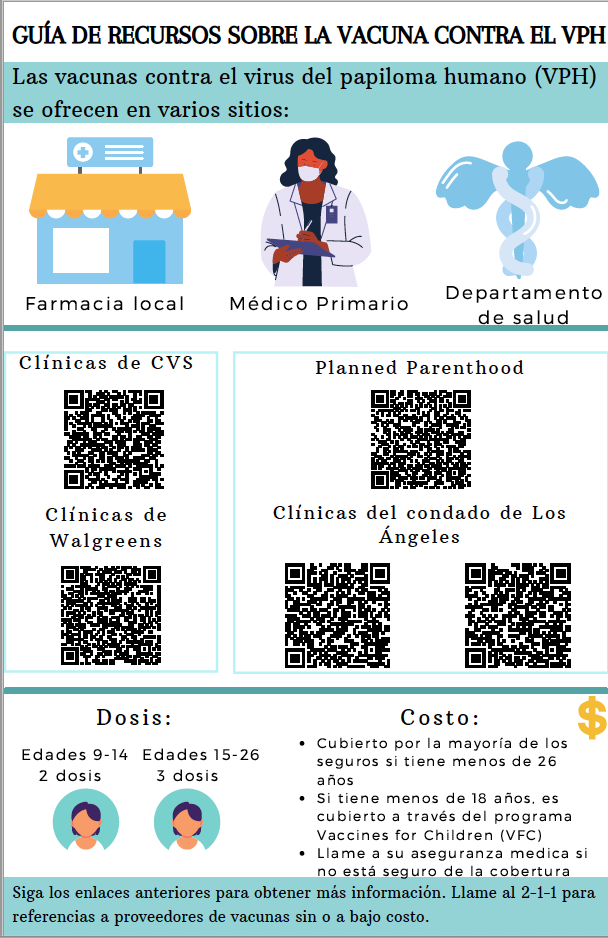


**Appendix B**

HPV Project Pre-Survey

1. Name
2. Level of Training
   1. PGY1
   2. PGY2
   3. PGY3
   4. PGY4
   5. Attending
3. I plan to use the laminated infographic to help me counsel patients about the HPV vaccine.
   1. Strongly Disagree
   2. Disagree
   3. Neutral
   4. Agree
   5. Strongly Agree
4. I plan to use the dot phrase to help me document HPV-related patient discussions.
   1. Strongly Disagree
   2. Disagree
   3. Neutral
   4. Agree
   5. Strongly Agree
5. The HPV resource-referral handout will encourage me to refer patients for the HPV vaccine.
   1. Strongly Disagree
   2. Disagree
   3. Neutral
   4. Agree
   5. Strongly Agree
6. I feel confident in my ability to counsel patients regarding HPV vaccination.
   1. Strongly Disagree
   2. Disagree
   3. Neutral
   4. Agree
   5. Strongly Agree
7. I feel up-to-date on current recommendations for HPV vaccination.
   1. Strongly Disagree
   2. Disagree
   3. Neutral
   4. Agree
   5. Strongly Agree
8. Educating women about preventing cervical cancer through HPV vaccination is a priority in our clinic.
   1. Strongly Disagree
   2. Disagree
   3. Neutral
   4. Agree
   5. Strongly Agree
9. I have experienced the following barriers when discussing HPV vaccination with my patients: (Please indicate the top 3 barriers you have experienced.)
   1. Lack of time
   2. Lack of provider knowledge
   3. Forget to mention
   4. Competing priorities in the clinic
   5. Unsure where to refer for vaccination
   6. Patient not interested in vaccination
   7. Patient has concerns about vaccination
   8. Other
10. Any recommendations for improving tools or increasing HPV vaccination in the clinic

HPV Project Post-Survey

1. Name
2. Level of Training
   1. PGY1
   2. PGY2
   3. PGY3
   4. PGY4
   5. Attending
3. I used the laminated infographic on the wall to help me counsel patients about the HPV vaccine.
   1. Always (for every patient <26)
   2. Frequently (at 75% of visits for patient's<26)
   3. Sometimes (at 50% of visits for patient's<26)
   4. Infrequently (at 25% of visits for patient's<26)
   5. Never
4. The laminated infographic was helpful when I counseled patients about the HPV vaccine.
   1. Strongly Disagree
   2. Disagree
   3. Neutral
   4. Agree
   5. Strongly Agree
5. I used the dot phrases to help me document HPV-related patient discussions.
   1. Always (for every patient <26)
   2. Frequently (at 75% of visits for patient's<26)
   3. Sometimes (at 50% of visits for patient's<26)
   4. Infrequently (at 25% of visits for patient's<26)
   5. Never
6. The dot phrases were helpful when documenting HPV-related patient discussions.
   1. Strongly Disagree
   2. Disagree
   3. Neutral
   4. Agree
   5. Strongly Agree
7. I received the HPV resource-referral handout with the green intake sheet for my patients <26
   1. Always (for every patient <26)
   2. Frequently (at 75% of visits for patient's<26)
   3. Sometimes (at 50% of visits for patient's<26)
   4. Infrequently (at 25% of visits for patient's<26)
   5. Never
8. The HPV resource-referral handout reminded me to refer patients for the HPV vaccine.
   1. Always (for every patient <26)
   2. Frequently (at 75% of visits for patient's<26)
   3. Sometimes (at 50% of visits for patient's<26)
   4. Infrequently (at 25% of visits for patient's<26)
   5. Never
9. I discuss HPV vaccination at postpartum visits
   1. Always (for every patient <26)
   2. Frequently (at 75% of visits for patient's<26)
   3. Sometimes (at 50% of visits for patient's<26)
   4. Infrequently (at 25% of visits for patient's<26)
   5. Never
10. I feel confident in my ability to counsel patients regarding HPV vaccination
    1. Strongly Disagree
    2. Disagree
    3. Neutral
    4. Agree
    5. Strongly Agree
11. I feel up-to-date on current recommendations for HPV vaccination.
    1. Strongly Disagree
    2. Disagree
    3. Neutral
    4. Agree
    5. Strongly Agree
12. Educating women about preventing cervical cancer through HPV vaccination is a priority in our clinic.
    1. Strongly Disagree
    2. Disagree
    3. Neutral
    4. Agree
    5. Strongly Agree
13. I have experienced the following challenges when discussing HPV vaccination with my patients: (Please indicate the top 3 challenges you have experienced. )
    1. Lack of time
    2. Lack of provider knowledge
    3. Forget to mention
    4. Competing priorities in the clinic
    5. Unsure where to refer for vaccination
    6. Patient not interested in vaccination
    7. Patient has concerns about vaccination
    8. Other
14. Any recommendations for improving tools or increasing HPV vaccination in the clinic:
